# Giardial lipid rafts share virulence factors with secreted vesicles and participate in parasitic infection in mice

**DOI:** 10.3389/fcimb.2022.974200

**Published:** 2022-08-23

**Authors:** Brian I. Grajeda, Atasi De Chatterjee, Carmen M. Villalobos, Breanna C. Pence, Cameron C. Ellis, Vanessa Enriquez, Sourav Roy, Sukla Roychowdhury, Aaron K. Neumann, Igor C. Almeida, Steven E. Patterson, Siddhartha Das

**Affiliations:** ^1^ Infectious Disease and Immunology, Border Biomedical Research Center and the Department of Biological Sciences, University of Texas at El Paso, El Paso, TX, United States; ^2^ Department of Pathology, School of Medicine, University of New Mexico, Albuquerque, NM, United States; ^3^ Center for Drug Design, University of Minnesota, Minneapolis, MN, United States

**Keywords:** *Giardia*, giardiasis, infection, lipid rafts, nystatin, oseltamivir, proteome, extracellular vesicles

## Abstract

*Giardia lamblia*, a protozoan parasite, is a major cause of waterborne infection, worldwide. While the trophozoite form of this parasite induces pathological symptoms in the gut, the cyst form transmits the infection. Since *Giardia* is a noninvasive parasite, the actual mechanism by which it causes disease remains elusive. We have previously reported that *Giardia* assembles cholesterol and GM1 glycosphingolipid-enriched lipid rafts (LRs) that participate in encystation and cyst production. To further delineate the role of LRs in pathogenesis, we isolated LRs from *Giardia* and subjected them to proteomic analysis. Various cellular proteins including potential virulence factors—e.g., giardins, variant surface proteins, arginine deaminases, elongation factors, ornithine carbomyltransferases, and high cysteine-rich membrane proteins—were found to be present in LRs. Since *Giardia* secretes virulence factors encapsulated in extracellular vesicles (EVs) that induce proinflammatory responses in hosts, EVs released by the parasite were isolated and subjected to nanoparticle tracking and proteomic analysis. Two types of EV—i.e., small vesicles (SVs; <100 nm, exosome-like particles) and large vesicles (LVs; 100–400 nm, microvesicle-like particles)—were identified and found to contain a diverse group of proteins including above potential virulence factors. Although pretreatment of the parasite with two giardial lipid raft (gLR) disruptors, nystatin (27 μM) and oseltamivir (20 μM), altered the expression profiles of virulence factors in LVs and SVs, the effects were more robust in the case of SVs. To examine the potential role of rafts and vesicles in pathogenicity, *Giardia*-infected mice were treated with oseltamivir (1.5 and 3.0 mg/kg), and the shedding of cysts were monitored. We observed that this drug significantly reduced the parasite load in mice. Taken together, our results suggest that virulence factors partitioning in gLRs, released into the extracellular milieu *via* SVs and LVs, participate in spread of giardiasis and could be targeted for future drug development.

## Introduction

*Giardia lamblia* is a waterborne parasite that affects an estimated 1.2 million people in the U.S. (Hunter and Thompson, 2005; Beer et al., 2017) and an approximately 280 million, worldwide (Plutzer et al., 2010). The *Giardia* infection or “giardiasis” is acquired through the ingestion of contaminated food and drinking water containing infective cysts. It has also been reported that pets can be reservoirs for this intestinal parasite. Studies from Portugal, India, and the U.S. reveal that giardiasis is a growing issue—along with a variety of other parasites—that could put pet owners and families at risk. (Ferreira et al., 2017; Utaaker et al., 2018; Duncan et al., 2020). When the cysts reach the stomach, they undergo excystation triggered by stomach acid (Roxström-Lindquist et al., 2006). The emerging excyzoites (i.e., newly excysted trophozoites) migrate downward into the duodenum and jejunum and adhere to the epithelium with the help of the ventral (adhesive) disc (Palm et al., 2005; Ankarklev et al., 2010).

Lipid rafts (LRs) are small nanoscale assemblies of cholesterol, sphingolipids (SLs), and proteins that form distinct liquid-ordered membrane domains resistant to extraction with nonionic detergents (Pike, 2009). In general, LRs participate in transducing extracellular signals downstream of the plasma membranes and can undergo self-assembly and disassembly (Lingwood and Simons, 2010). These membrane microdomains also participate in host-pathogen interactions. For example, LRs facilitate the entry of viruses, bacteria, and other intracellular pathogens into host cells (Kulkarni et al., 2021). Many intracellular pathogens can induce the clustering of receptors and proteins present in LRs to trigger downstream signaling effects (Simons and Ikonen, 1997). Likewise, host-cell LRs can participate in mounting anti-viral and anti-bacterial responses (Kulkarni et al., 2021). In fungal and protozoan cells, LRs are responsible for determining their virulence (Rella et al., 2016). In *S. cerevisiae*, ATPase-1 and glycosylphosphatidylinositol (GPI)-anchored proteins are in the LR microdomains and translocate to vacuoles from membranes. (Bagnat et al., 2001; Santos-Pereira et al., 2021), suggesting that the raft formation is important for maintaining the topology of membrane proteins in yeast cells. Monosialodihexosylganglioside (GM3)-enriched raft domains are present in endoplasmic and cytoplasmic faces of *Plasmodium falciparum* and are thought to be linked to protein-transport events in infected red blood cells (Koudatsu et al., 2021). Monosialotetrahexosylganglioside (GM1) and GM3, two major glycosphingolipid components of LRs in *Toxoplasma gondii*, are scavenged by the parasite from infected host cells (Konishi et al., 2021). Phosphatidylinositol 4,5-bisphosphate [PI(4,5)P_2_ or PIP_2_] in *Entamoeba histolytica* is located in raft domains and regulates the cellular motility of this eukaryotic pathogen (Koushik et al., 2013).

The presence of cholesterol and GM1-enriched LRs has also been identified in *Giardia* (De Chatterjee et al., 2015). In trophozoites, these microdomains are assembled mostly in plasma membranes. In cysts, LRs are located in membranes beneath the cyst wall and in the cytoplasm. Cholesterol-binding compounds and GM1 synthesis/transport inhibitors have been found to block LR formation in *Giardia*. For instance, nystatin and filipin III, two cholesterol-binding agents, and oseltamivir (Tamiflu^®^), a viral neuraminidase inhibitor, disassembled the microdomains and inhibited encystation in culture. Detergent-resistant and GM1-/cholesterol-enriched LRs were isolated from *Giardia* by density-gradient centrifugation and found to be sensitive to nystatin and oseltamivir (De Chatterjee et al., 2015).

In recent years, efforts have been made to understand the roles of giardial extracellular vesicles (GEVs) in modulating the host immune system (Zhao et al., 2021a; Zhao et al., 2021b). There are three types of extracellular vesicles (EVs), each emanating from different cellular sites including the plasma membranes. Exosomes are smaller vesicles that range in size from 40 to 100 nm and are released after the fusion of multivesicular bodies to the plasma membrane (Colombo et al., 2014; Skotland et al., 2017). Microvesicles are larger than exosomes that range in size from 100 to 1000 nm (Colombo et al., 2014). Finally, there are apoptotic bodies that range in size from 500 to 2000 nm and emerge from apoptotic cells (György et al., 2011; Raposo and Stoorvogel, 2013). Reports have also suggested that host cells induce the release of metabolic enzymes/proteins from *Giardia*, and these proteins (known as the “giardial virulence factors”) have altered the innate immune defense system of the host epithelial cells in culture (Ringqvist et al., 2008; Skarin et al., 2011; Ma’ayeh et al., 2017). However, the attachment of trophozoites to host-epithelial cells is not a prerequisite for the secretion of virulence factors (Emery et al., 2016). Secreted enzymes and proteins (secretome) released by *Giardia* include arginine deaminase (ADI), carbamate kinase (CK), giardins, variant surface proteins (VSPs), elongation factors (EFs), ornithine carbamoyl transferases (OCTs), high cysteine-rich membrane proteins (HCMPs), tenascins, and proteases (Dubourg et al., 2018; Ortega-Pierres and Argüello-García, 2019). Microvesicles released by *Giardia* are likely to contain a majority of these virulence factors, which have been shown to be absorbed by human immature dendritic cells, resulting in immune responses (Evans-Osses et al., 2017). Zhao et al. (2021a) reported that EVs released by trophozoites (giardial EVs or gEVs) are captured by macrophages, induce immune responses, and stimulate the production of proinflammatory cytokines along with Toll-like receptor 2 (TLR2) and NOD-like receptor family pyrin domain-containing 3 (NLRP3) inflammasome-signaling pathways. The authors have also demonstrated that the signaling cascade of p38 MAPK, p44/42 MAPK (Erk1/2), AKT, and NF-κB are affected in macrophages by gEVs (Zhao et al., 2021b). These studies have demonstrated the pivotal roles of gEVs that damage host cells during host*-Giardia* infections.

In the current study, we demonstrate that: (1) *Giardia* has the ability to form LRs at the nanoscale level, and many virulence factors are partitioned into these raft domains; (2) two types of EVs (SVs and LVs) are produced by *Giardia* and share many proteins including the virulence factors with LRs; (3) repurposed drugs such as nystatin and oseltamivir, which disassemble LRs in *Giardia* (De Chatterjee et al., 2015), also affect the size and protein-distribution patterns in SVs rather than LVs; (4) finally, we show that oseltamivir is effective in reducing *Giardia* infectivity in mice, suggesting a clear link between raft assembly, vesicle biogenesis, and infection caused by this worldwide waterborne pathogen that is particularly prevalent in countries with lower resources.

## Materials and methods

Most chemicals used in this investigation were purchased from Sigma-Aldrich (St. Louis, MO) with the exceptions listed below. Solvents and reagents used for mass spectrometry were of LC-MS grade from Thermo Fisher Scientific (Waltham, MA). Adult bovine serum (catalog no. SH30075.03) was obtained from Hyclone Laboratory (Utah). Cyst antibody was purchased from Santa Cruz (Santa Cruz, CA) and Invitrogen (Carlsbad, CA), respectively. Lipid-raft labeling kit (Vybrant Alexa Fluor 488, V34403) was purchased from Invitrogen (Carlsbad, CA). GM1 antibody (polyclonal) was obtained from Abcam (Waltham, MA), and Nystatin was purchased from Sigma-Aldrich. Oseltamivir (Tamiflu^®^) and myriocin were purchased from Selleckchem (Houston, TX) and Sigma-Aldrich, respectively.

### Cell culture

*Giardia lamblia* trophozoites assemblage A (strain WB C6; ATCC No. 30957) and assemblage B (strain GS H7; ATCC 50581) were cultivated using modified TYI-S-33 medium supplemented with 10% adult bovine serum and 0.5 mg/mL bovine bile (Diamond et al., 1978; Keister, 1983). The antibiotic piperacillin (100 µg/mL) was added during routine culturing of the parasite (Gillin et al., 1989). Growth was initiated by adding ∼10^5^ trophozoites/mL in the culture medium and continued to grow until the cells became 80–90% confluent (∼48 h). To produce cysts, trophozoites from WB isolates were cultured in high bile medium, as previously described by Keister (1983). The trophozoites and cysts forms of human *Giardia*-isolate H3 (assemblage B), collected from gerbils, were purchased from Waterborne Inc. (New Orleans, LA), and HT29 cells lines were obtained from the ATCC.

### Identification and isolation of lipid rafts

LRs from *Giardia* trophozoites and cysts were identified by immunostaining with cholera toxin B (CTXB) and GM1 antibody. Briefly, cells were harvested by centrifugation, washed in PBS, and stained with CTXB or GM1 antibody as previously described (De Chatterjee et al., 2015). For CTXB labeling, _~_1x10^7^ cells (trophozoites and cysts) were fixed in 4% formaldehyde, followed by immunostaining with CTXB conjugated to Alexa Fluor 488 (1 μg/mL) for 10 min, and labeling with CTXB antibody (1:200 dilution) for 15 min, as recommended by the manufacturer. LRs were also immunostained with GM1 antibody. For this, cells were incubated overnight with GM1 antibody (1:50 dilution) in cold (6-10°C) and then with a secondary antibody conjugated to Alexa Fluor 568 (1:500 dilution) for 1 h, at room temperature (RT). Both CTXB- and GM1-labeled cells were mounted with ProLong Gold antifading reagent with DAPI (Invitrogen) and visualized by confocal microscopy (Carl Zeiss LSM 700 confocal microscope). For the drug treatments, trophozoites were incubated with nystatin (27 μM), oseltamivir (20 μM), and myriosin (27 μM) for 30 min, at 37°C, before staining CTXB and GM1 antibody, as described by De Chatterjee et al. (2015).

LRs from *Giardia* (after treatment with nystatin and oseltamivir) were isolated following the method described by De Chatterjee et al. (2015). Briefly, ~1x10^9^ trophozoites were collected from the TYI-S-33 growth medium (supplemented with bovine serum and bile), washed in PBS, and incubated with HRP-conjugated CTXB for 1 h at 4°C (Blank et al., 2002; Graham, 2002). Trophozoites were resuspended in 5 mM Tris-HCl buffer (pH 7.8) containing EDTA (2 mM), DTT (0.4 mM), Triton X-100 (1%), and Halt Protease Inhibitor Cocktail (Thermo Fisher Scientitific). Lysate was placed at the bottom of Optiprep™ density gradient (35, 30, 25, 20, and 0%). Samples were centrifuged at 200,000 x*g* for 4 h, in a Sorvall T-865.1 fixed-angle rotor (Sorvall WX Ultra Series Centrifuge; Thermo Fisher Scientific, IL). Fractions of 1 mL were collected from the top of the gradient. To investigate which LR fractions were enriched in cholesterol and GM1, a cholesterol measurement kit (Invitrogen) and GM1 antibody-based ELISA assays were employed as described by De Chatterjee et al. (2015). Cholesterol- and GM1-enriched-LR fractions were subjected to proteomic analysis, as described below.

### Isolation of extracellular vesicles

Large and small extracellular vesicles (EVs) from *Giardia* trophozoites were isolated by the method described by Gavinho et al. (2020). Briefly, *Giardia* cells were grown to confluency in 75-cm^2^ flasks, as described above. Approximately 1 x 10^7^ trophozoites were harvested *via* an ice chill, and the cells were then pelleted by centrifugation at 2,400 x*g*, for 5 min at 4°C. For the drug treatments, cells were resuspended in TYI-S-33 medium (pH 7.1) without serum that contained nystatin (27 μM), oseltamivir (20 μM), and myriocin (27 μM) for 30 min at 37°C, followed by centrifugation at 2,400 x*g* at 4°C, for 5 min. The trophozoites (~1x10^7^ cells) were washed three times in PBS and resuspended in the incubation buffer (PBS containing 5 mM L-cysteine, 5 mM glucose, and 1 mM CaCl_2_, pH 7.1) and incubated for 3 h at 37°C to allow the secretion of EVs. The viability was checked every hour by staining with propidium iodide and flow cytometry (Cytomics FC500; Beckman Coulter) analysis (**Figure S1**) following the protocol from Ruiz-Medina et al. (2019). Cell debris were removed by concurrent low speed centrifugation at 600 xg (5 min) and 4000xg (30 min) at 4°C, respectively. Vesicles were isolated by differential centrifugation; large vesicles (LVs, microvesicle-like) were collected from the supernatant after 15,000 x*g* (60 min) at 4°C and small vesicles (SVs, exosome-like) were collected from pellet after centrifugation at 100,000 x*g* for 4 h, at 4°C (Gavinho et al., 2020).

### Nanoparticle tracking analysis

After treatment with nystatin and oseltamivir, the size (diameter) and number of EVs in each EV-enriched fraction were measured by nanoparticle tracking analysis (NTA), using a NanoSight Model LM14C (Malvern Panalytical Ltd., Malvern, UK) (Bayer-Santos et al., 2013). Samples were diluted 1:10 in triple-filtered PBS. NTA 3.2 Dev Build 3.2.16 software was used for the analysis, with the following settings: 30 sec capture for a total of three captures, Camera Level: 14, Slider Shutter: 1259, Slider Gain: 366, FPS 25.0. Four independent experiments in triplicate were conducted, and the results were represented by bar graphs with appropriate statistical analysis. Representative figures directly acquired from the NanoSight are also shown in the results section.

### dSTORM microscopy

*Giardia* samples were fixed in paraformaldehyde (PFA, 4%) at 4°C for 10 min, followed by vigorous washing. For LR staining, ~3x10^6^ trophozoites/mL were immunostained with CTXB-AF647 conjugate (dilution 1:1000) (Thermo Fisher Scientific, C34778) at 4°C for 10 min, followed by washing in PBS. The cells were then treated for 15 min at 4°C with a 1:200 dilution of anti-CTXB and 1% normal goat serum for 5 min. Cells were washed and samples were embedded in 4% low-gelling temperature agarose (Sigma-Aldrich, A9414) on a coverslip. Imaging buffer (50 mM Tris, 10% glucose, 10 mM NaCl, 40 mg/mL catalase, 500 mg/mL glucose oxidase and 10 mM cysteamine) (Sigma-Aldrich, M9768) was administered to the agarose-embedded samples for 15 min prior to capturing images following the Lin et al. (Lin et al., 2016) method. Data was collected using an oil-immersion objective (PlanApo N, 150x/1.45 NA; Olympus) in an oblique illumination setup on an Olympus IX-71 microscope outfitted with an objective-based TIRF illuminator. A 637-nm laser (Thorlabs, laser diode HL63133DG) with custom-built collimation lenses was used to excite the samples. A self-registration technique was used to ensure that minimal drift occurred during data collecting (Graus et al., 2018). We utilized a maximum likelihood-based technique from (Smith et al., 2010) to fit probe positions from raw dSTORM data. As reported in Graus et al. (2018), we used normal fitting with a Cramer-Rao lower bound (CRLB)-based, fit-accuracy threshold of 0.2 pixels or 22 nm.

Cluster analysis: the hierarchical single emitter hypothesis (H-SET) was used to estimate the placement of single emitter probes and reduce errors in subsequent cluster identification processes, as reported by Graus et al. (2018). The first H-SET pass condensed clusters of observations of several blinking fluorophores into single estimates of real fluorophore localizations. The second H-SET pass completed any multiple localization-collapse procedures left incomplete in the first pass and applied a density-based spatial clustering of applications with noise cluster-identification technique (DB-SCAN) (Ester et al., 1996). DB-SCAN has two parameters: epsilon, a distance metric that measures the maximum distance between two points within a cluster, and minpoints, the minimum number of points needed to form a cluster. By running 100 H-SET iterations over a variety of epsilons and minpoints, these values were optimized. The epsilon and minpoints values used for the analysis of the *G. lamblia* images were 29 nm and 3 points, respectively. Singlet clusters are defined as one or two localizations and multi clusters are defined as three or more.

### Transmission electron microscopy

For transmission electron microscopy (TEM) analysis, samples were fixed in 2% paraformaldehyde/2.5% glutaraldehyde in 100-mM sodium cacodylate buffer for 2 h at room temperature. The samples were then embedded in 2.5% agarose and post-fixed in 2% osmium tetroxide (Ted Pella Inc. Redding, CA) for 1 h at RT. After three washes with deionized water, samples were stained en bloc in 1% aqueous uranyl acetate (Electron Microscopy Sciences, Hatfield, PA) for 1 h. Samples were then rinsed in distilled water, dehydrated in a graded series of ethanol, and embedded in Eponate 12 resin (Ted Pella Inc). The samples were cut into 95-nm sections with a Leica Ultracut UCT ultramicrotome (Leica Microsystems Inc., Bannockburn, IL), stained with uranyl acetate and lead citrate, and viewed on a JEOL 1200 EX transmission electron microscope (JEOL USA Inc., Peabody, MA) equipped with an AMT 8-megapixel digital camera and AMT Image Capture Engine V602 software (Advanced Microscopy Techniques, Woburn, MA). Released vesicles were isolated by the method of Evans-Osses et al. (2017) and examined by TEM. Briefly, trophozoites were resuspended in the incubation buffer and incubated for 3 h at 37°C as described above. Cells and debris were separated by centrifugation at 600 xg (5 min) and 4000 x *g* (30 min) at 4°C, respectively. Vesicles (containing LVs and SVs) were collected and mixed with glutaraldehyde (1% final concentration). Samples were then allowed to absorb onto freshly glow discharged formvar/carbon-coated copper grids for 10 min. Grids were then washed in dH_2_O and negative stained with 1% aqueous uranyl acetate (Ted Pella Inc.) for 1 min. Excess liquid was gently wicked off and grids were allowed to air dry. Samples were viewed on a transmission electron microscope.

### Proteomic analysis of giardial EVs

The large and small vesicles (LVs and SVs) secreted by *Giardia* were isolated by differential density- gradient centrifugation, as described above, and subjected to proteomic analysis by one-dimensional liquid chromatography-tandem mass spectrometry (1D-LC-MS/MS) (Szempruch et al., 2016; Ribeiro et al., 2018; Cronemberger-Andrade et al., 2020; Cortes-Serra et al., 2020). Briefly, 1 μL of digested peptides was injected into a custom packed AQUA 5μm,125Å, C18 (Phenomenex, cat no. 04A-4299) 20 cm, 15 ± 1 μm, PicoFritEmitter (New Objective) equilibrated with optima grade (Thermo Fisher Scientific) 5% solvent B (90% acetonitrile, 0.1%formic acid), and 95% solvent A (0.1% formic acid). Peptides were applied and washed for 10 min continuously with 5% solvent B at a flow ratio of 0.5 μL/min maintained throughout the run before applying the elution gradient. Solvent B was increased to 35% over 85 min, and then increased to 95% over 5 min to maintain a high organic plateau for 9 min. The elution was decreased to 5% solvent B for 1 min and maintained at 5% solvent B for re-equilibration prior to the next sample injection for a total runtime of 120 min. The mass spectrometer was equipped with a NanoSpray Flex ion source (Thermo Fisher Scientific). Peptides were analyzed in a top 10 data-dependent MS2 acquisition by a Q-Exactive Plus Hybrid Quadrupole-Orbitrap mass spectrometer (Thermo Fisher Scientific). Full scan parameters were set to resolution of 70,000, 3e6 AGC target, and a scan range of 350 to 1600 *m/z*. MS2 parameters were set to 17, resolution of 500,1e5 AGC target, isolation window at 3.0 *m/z*, (N) CE: 30, charge exclusion: unassigned, 1, >8.

### Proteomic analysis of giardial lipid rafts

A proteomic analysis of gLRs was carried out by two-dimensional (2D) LC-MS/MS. All experiments were conducted with a U300 Q-Executive mass spectrometer and Ultimate 3000 RS HPLC System. A 10-h, 5-step 2D-LC-MS/MS method was run with 5-µl injections of ammonium acetate from the auto sampler (100, 250, 500, 750, and 1000 mM). One-hundred-fifty micromolar fused silica was used and packed with 5 cm of SCX material (Luna). We used the following instrumental conditions: top 10, runtime: 120 min, default charge state: 2, resolution: 70,000, AGC target: 3e6, maximum IT: 100ms, scan range 400 to 1600 *m/z*, for dd-MS2 resolution: 17,500, AGC target 1e5, maximum IT: 50 ms, Minimum AGC target: 8e3, dynamic exclusion: 15s. Solvent A was 95% acetonitrile, 5% water, and 0.01% formic acid. Solvent B was 5% water, 95% acetonitrile, and 0.01% formic acid. Solvent C was 500 mM ammonium acetate in solvent A. Run gradients were (t=0-5min, 5% B; t=5-5.1 min, per pulse [20, 40, 60, 80, and 100%] C; t=5.1-7.5 min, [20, 40, 60, 80, and 100%] C; t=7.5-8 min, 5% B; t=8-15 min, 5% B; t=15-120 min, 50% B). Wash gradients (t=0-5 min, 5% B; t=5-20 min, 95% B; t=20-22 min, 5% B; t=22-25 min, 5% B; t=25-40 min, 95% B; t=40-42 min, 5% B; t=42-60 min, 5%) were performed with a flow rate of 300 µL/min.

### Bioinformatics

Proteome Discover (PD) 2.5.0.400 (Thermo Fisher Scientific) was utilized to process the raw files from the 1D- and 2D-LC-MS/MS analyses. Database for *Giardia* (30,894 sequences) was downloaded from UniProtKB (http://www.uniprot.org/) on 21 April, 2021. A contaminant data set composed of trypsin autolysis fragments, keratins, and standards found in the CRAPome (Mellacheruvu et al., 2013) repository and in-house contaminants were run in parallel. The PD analysis parameters were as follows: a false-discovery rate (FDR) of 1%, HCD MS/MS, fully tryptic peptides only, up to 2 missed cleavages, a parent-ion mass of 10 ppm (monoisotopic); fragment mass tolerance of 0.6 Da (in Sequest) and 0.02 Da (in PD 2.1.1.21) (monoisotopic). Two-high confidence peptides per protein were applied for identification. The PD dataset was processed through Scaffold Q+ 4.8.2 (Proteome Software, Portland, OR) for protein quantification. A protein threshold of 99%, peptide threshold of 95%, and a minimum number of 2 peptides were used for protein validation.

### Attachment assay

To conduct the *Giardia* attachment assay, HT-29 (human adenocarcinoma cell line) cells were grown in a four-well chamber slide (Nunc 155383 Chambered Cover Glass, Thermo Fisher Scientific) in McCoy’s medium supplemented with 10% fetal bovine serum (FBS). *Giardia* trophozoites were cultured in regular growth medium supplemented with bile and adult bovine serum (Gillin et al., 1989) described in Materials and Methods. Cells were pre-treated with one of the following inhibitors—i.e., nystatin (27 μM), oseltamivir (20 μM), methyl-β-cyclodextrin (MBCD, 1 mM) or myriocin (27 μM) for 30 min at 37°C. Attached trophozoites were harvested by chilling the flasks in icy water, collected by centrifugation (2,500 xg), washed in ice-cold PBS, and added (~0.5x10^7^ trophozoites) to the confluent monolayer of HT-29 cells. *Giardia* trophozoites and HT-29 cells were co-incubated for 3 h at 37°C in incomplete McCoy’s medium. Slides were removed from the chambers, washed, fixed in 4% paraformaldehyde, and incubated with TSA 417 antibody (TSA417, polyclonal, 1:100 dilution- obtained from Dr. Frances Gillin, UCSD; Gillin et al., 1990) followed by reacting with anti-rabbit secondary antibody conjugated to Alexa fluor. DAPI was used to stain the DNA of HT29 cells. Attached trophozoites were

### Mouse experiments

Mouse experiments were performed under the vertebrate animal protocol approval of the Institutional Animal Care and Use Committee (IACUC) of the University of Texas at El Paso. Briefly, 3-4-week-old mice (C57BL/6) were given a cocktail of three antibiotics—i.e., ampicillin, neomycin (G418), and vancomycin following the methodologies described earlier by Solaymani-Mohammadi and Singer (2011) and Bartelt et al. (2013). All antibiotics were given at a concentration of 1mg/mL ad libitum by mixing with drinking water. The antibiotic treatment was initiated three days (72 h) prior to infecting the mice with *Giardia* and continued throughout the experiment. C57BL/6 mice (5 per group) were then infected with ~1 x 10^7^ luciferase-expressing WB trophozoites (resuspended in 0.1 mL PBS) to establish the infection. The firefly luciferase expressing PNT5 construct (Pan et al., 2009) was obtained from Dr. Chin-Huang Sun (Taiwan). This plasmid (PNT5) originated from 5’Δ5N-pac construct prepared by Dr. Steve Singer and his colleagues (Singer et al., 1998). The firefly luciferase gene was inserted at the NcoI/EcoRI sites. The resulting plasmid, PNT5-Luc+, contained the luciferase gene under the control of the α2-tubulin promoter (Pan et al., 2009). *G. lamblia* (WB isolate, assemblage A) was transfected with PNT5-Luc+, selected under neomycin and gavaged into mice. The treatment was initiated with oseltamivir after 48 h of infection. The doses of oseltamivir were 1.5 and 3 mg/kg per mouse, respectively. Drugs were resuspended in 0.1 mL PBS and gavaged every day for 13 days. Metronidazole (3.0 mg/kg) was used as a positive control. Mice were imaged through IVIS Lumina III *In Vivo* Imaging System (Perkin Elmer) to track the colonization of trophozoites in the intestine, and the parasite load was monitored by counting cysts collected from fecal materials. The method of sucrose floatation technique described by Roberts-Thomson et al. (1976) was used (with some modifications) to isolate cysts. Briefly, ~200 mg of fecal material from each mouse was collected and resuspended in 2.5mL of sterile PBS and subjected to homogenization. The fecal suspension was layered on 3mL sucrose solution (0.85M). The samples were centrifuged at 1,200 rpm for 10 min at 4°C. Cysts were collected from the water-sucrose interface with a Pasteur pipette (~700 μL) and counted under a microscope with the help of a hemocytometer.

## Results

### Organization and size determination of lipid rafts in *Giardia*

**Figure 1:**
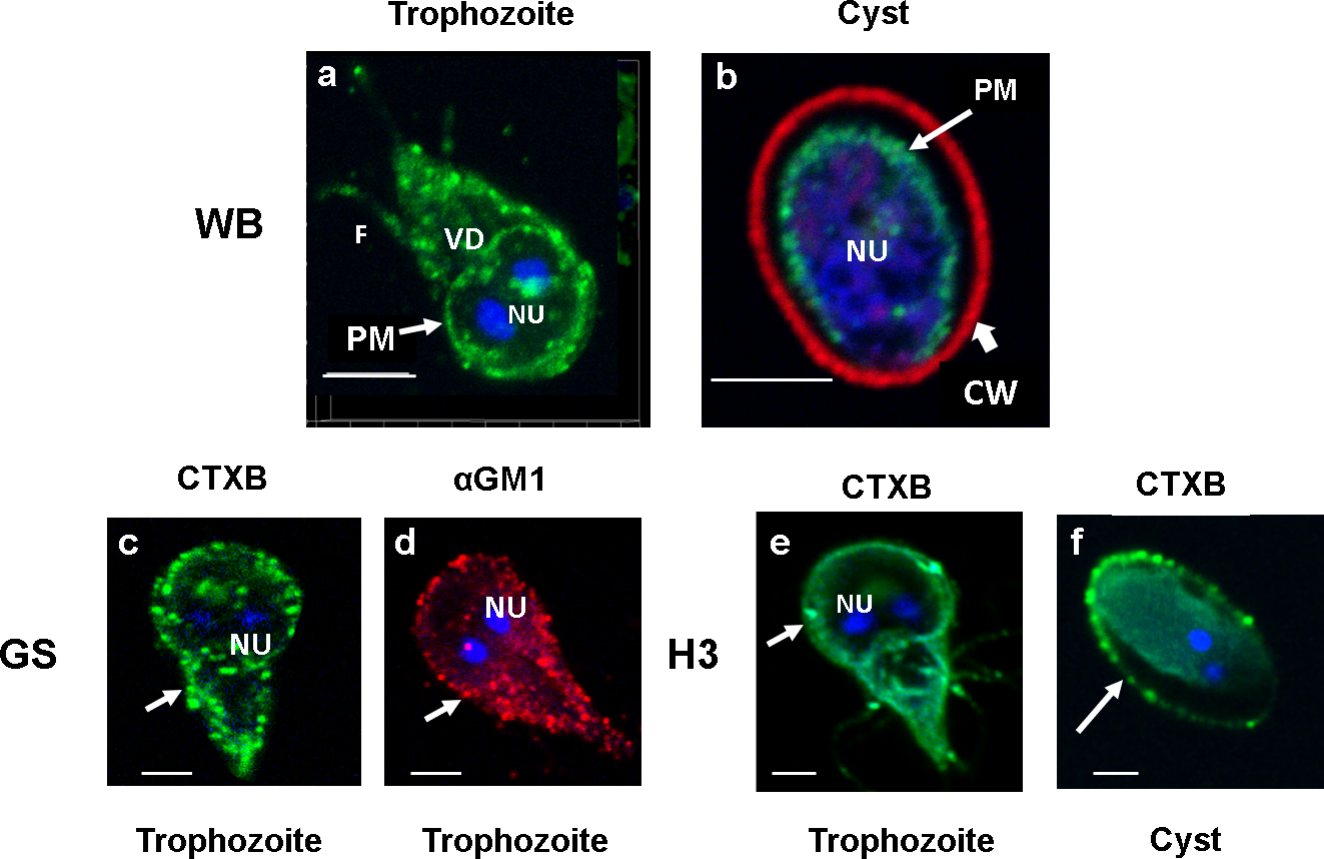
Panel **(A)**. Staining of lipid rafts in WB, GS and H3 isolates of *Giardia*. Trophozoites were labeled with Alexa Fluor (488)-conjugated CTXB (images **A**, **C** and **E**) and GM1 antibody (image D) Immunostaining of raft domains in plasma membranes is visible. DAPI-stained nuclei (NU) are also visible. The image of CTXB-labeled *Giardia* trophozoites and cysts (images **B**, **F**) were captured with the help of a confocal microscope (Carl Zeiss LSM 700) and analyzed by Zen 2009 software. Z-stacks were acquired, and a 3D model was reconstructed from the 12 optical sections of the z-stacks with a slice thickness of 0.37 μm each. Bars, 5 μm.

Lipid rafts (LRs) are cholesterol- and sphingolipid (SL)-enriched nanoscale compartments of membranes that participate in various biological processes. We have previously shown that gLRs are enriched in cholesterol and monosialoganglioside 1 (GM1) (De Chatterjee et al., 2015). In the current experiment, to visualize raft domains, we used fluorescently conjugated cholera toxin B (CTXB). CTXB binds with GM1 glycolipids (Blank et al., 2002), and therefore, it is conceivable that GM1-enriched membrane domain should react with CTXB. The confocal microscopy analysis (**Figure 1** **)** reveals that the trophozoite plasma membranes immunostained with Alexa fluor 488-conjugated CTXB. It is also clear from the images that staining with CTXB produces green punctate-raft-like structures in the membranes of trophozoites (WB isolate). In addition, the periphery of the ventral disc (VD) of WB isolate (trophozoites) also reacts faintly with CTXB (**Figure 1** **A**) (De Chatterjee et al., 2015), indicating the presence of small amount of membrane components (especially the GM1 glycolipid) could be associated with the VD (Chavez and Martinez-Palomo, 1995). LRs are present in the plasma membrane of *in vitro*-derived, water-resistant cysts (WB isolate). The immunostaining of cysts with CTXB (green) and cyst-wall protein (CWP) antibody (conjugated with Alexa Fluor 568) shows the presence of raft domains in the cyst plasma membrane beneath the cyst walls (red) (**Figure 1** **B**). Rafts are also present in GS and H3 isolates, and they all show the same punctate structures (**Figures 1** **C, E**). However, the staining of VD with CTXB is not noticeable in these isolates. **Figure 1** **D** shows the staining of LRs in GS isolate (trophozoite) with GM1 antibody conjugated with Alexa Fluor 568 (red), which shows a similar labeling pattern with CTXB (**Figure 1** **C**), supporting the observation that CTXB binds with GM1 lipid of gLRs. (**Figure 1** **F**) is the staining of *in vivo*-derived cysts (H3 isolate) collected from gerbils. Interestingly, in this cyst, LRs are associated with the plasma membrane as well as the endomembranes concentrated in the cytoplasm. Here, no attempt was made to stain the cyst wall and therefore the wall not visible in the figure (**Figure 1** **F**).

**Figure 2:**
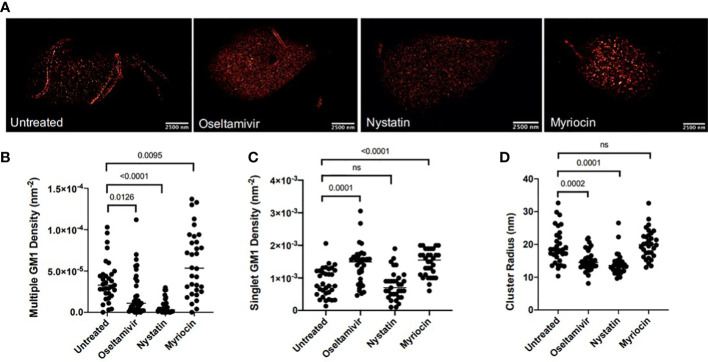
Nanoscopic GM1 clustering characteristics. **(A)** Representative dSTORM images of *G lamblia* untreated and treated with various agents. Bars, 2500nm **(B)** Hierarchical clustering quantification of nanoscopic GM1 cluster geometries for densities of multiple GM1 clusters. **(C)** Hierarchical clustering quantification of nanoscopic GM1 singlet densities. **(D)** Quantification of GM1 cluster radii. 15 cells were imaged per sample, and regions of interest (ROI) were selected on each cell. Each sample ranges from 32-35 total ROIs investigated. ns, non significant.

Because of their dynamic nature, the size of LRs in the eukaryotic cells can vary from 10 to 200 μm (Day and Kenworthy, 2015). Since *Giardia* is an early-diverging eukaryote and gLRs participate in encystation and cyst production (De Chatterjee et al., 2015), we wanted to determine the size of raft domains in this parasite. We also asked if nystatin and oseltamivir could be effective in disassembling gLRs at the nanoscale range. We employed single molecular localization-based super-resolution microscopy. More specifically, an analysis was done with direct stochastic-optical reconstruction microscopy (dSTORM). Single-molecule-based, super-resolution techniques use photo-switchable fluorophores, single-molecule localization, temporal separation, and image reconstruction, which achieved an optical resolution of down to ~20 nm in the imaging plane (Betzig et al., 2006; Heilemann et al., 2008; Heintzmann and Huser, 2017). dSTORM imaging identified CTXB-labeled membrane structures consisting of multiple (≥3) CTXB labels. These structures were termed “multiclusters” and are interpreted as representative of gLRs. The mean radius of CTXB-labeled multiclusters in untreated *Giardia* was 19.29 nm, as shown in **Figure 2D**. **Figure 2A** shows the staining of GM1-enriched gLRs by CTXB and the overall distribution of raft sizes after treatment with nystatin and oseltamivir. Giardial cells were treated with the LR disruptors—nystatin (27 μM) and oseltamivir (20 μM)—to determine any changes in the LR-size distribution. Myriocin (27 μM), an inhibitor of 3-keto-sphinganine synthesis (Chen et al., 1999), was used as a nonspecific binding reference (De Chatterjee et al., 2015). When cells were treated with LR disruptors, the multicluster density of GM1-enriched LRs significantly decreased following treatment with oseltamivir or nystatin (**Figure 2B** **)**. Subsequently, the mean density of singlet GM1 clusters increased in oseltamivir-treated samples but not with nystatin (**Figure 2C**). At this point, it is not clear why nystatin that disrupts multiple GM1 densities (**Figure 2A**) fails to influence the singlet GM1 density. **Figure 2D** shows the mean cluster radius of samples treated with either LR disruptor has significantly decreased. This provides evidence that treatment with oseltamivir and nystatin affects the size and distribution of LR microdomains in *Giardia*.

### Raft disruptors block the attachment of trophozoites to host cells and affect EV secretion

*Giardia* attachment on host intestinal cells is critical for establishing replication, producing intestinal infection, and causing subsequent giardial encystation or cyst formation. We have previously reported that two repurposed drugs—i.e., nystatin and oseltamivir—inhibit cyst production by disrupting the LRs (De Chatterjee et al., 2015). Studies also indicate that MBCD, a cholesterol-binding agent and a common LR inhibitor, blocks the attachment of trophozoites on cultured Caco-2 cells (Humen et al., 2011), implicating that giardial attachment to host cells is likely to be linked to gLRs. Attachment through LRs has also been reported in case of human sperm cells on oocytes (Van Blerkom and Caltrider, 2013), and evidence suggests that LRs regulate bacterial attachment and viral entry into host cells (Chatterjee et al., 2021; Palacios-Rápalo et al., 2021). It been demonstrated that, MBCD blocks microvesicles biogenesis as well as attachment to CaCo-2 cells by *Giardia* (Evans-Osses et al., 2017), further supporting the idea that LRs, giardial attachment, and vesicle biogenesis are interdependent. In the current experiment, we investigated whether two repurposed drugs—i.e., nystatin and oseltamivir—inhibit trophozoite attachment on cultured intestinal epithelial cells. We also asked about the types and classes of EVs secreted by *Giardia*, and whether these two compounds interfere with the secretion processes.

### (i) Nystatin and oseltamivir inhibit the attachment of *Giardia* on cultured HT29 cells

**Figure 3:**
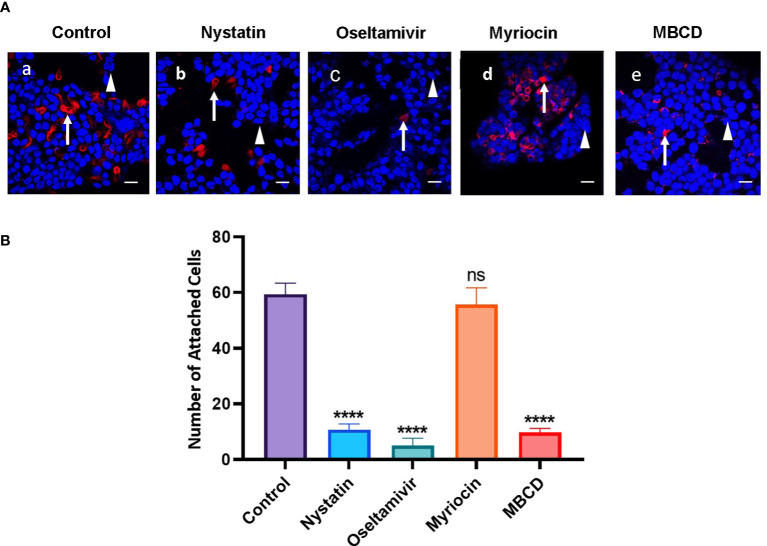
Raft inhibitors block the attachment of trophozoites to HT29 cells. **(A)** Trophozoites were incubated for 30 min at 37°C in the presence of nystatin (27 μM), oseltamivir (20 μM), myriocin (20 μM) and MBCD (1mM) as described in Materials and Methods. Treated trophozoites were collected, washed in PBS, counted, and added to a confluent monolayer of HT29 cells. The *Giardia* and HT29 cells were co-incubated in serum-free McCoy’s medium (pH, 7.4) for 3 h at 37°C. After removing the McCoy’s medium, co-cultured cells (HT29 and trophozoites) were fixed, labeled with antibodies against a major surface protein of *Giardia* (TSA 417) conjugated to Alexa Fluor 568) (Gillin et al., 1990), stained with DAPI (to identify the nucleus of HT29 cells), and analyzed by were captured with the help of a confocal microscope (Carl Zeiss LSM 700) and analyzed by Zen 2009 software. Arrow indicates trophozoites (red) and arrowhead shows the DNA of HT29 cells (blue). Bar: 2 μm. **(B)** For quantitation, attached trophozoites (red) were counted to observe the effects inhibitors. Approximately, 50-100 co-cultured cells were examined from 5-10 different fields to identify attached cells. The results were presented in mean values of attached cells ± SD of three separate experiments conducted in different days with different samples preparations. ****P<0.001; ns, not significant.

Since the attachment of *Giardia* on the small intestine lumen is critical for establishing gut infection (Einarsson et al., 2016), and because MBCD (an LR inhibitor) inhibits the attachment of trohozoites to Caco-2 cells (Humen et al., 2011), we investigated whether nystatin and oseltamivir, which disassemble raft domains (De Chatterjee et al., 2015), also inhibit the attachment of *Giardia.* To conduct this experiment, HT29 (human colorectal adenocarcinoma) cells were cultured in a four-well chamber slide as described in the methods section. Nystatin, oseltamivir, myriocin, and MBCD were added onto the attached trophozoites, as described by De Chatterjee et al. (2015) and Humen et al. (2011). Myriocin was used as a negative control and MBCD as a positive raft disruptor. The treatment was carried out for 30 min. Cells were collected washed in PBS, added onto the confluent monolayer of HT29 cells, and allowed to co-incubate for 3h at 37°C. The washing of inhibitor-treated trophozoites minimized the chances of residual compounds that could affect the LRs of HT29 cells (Humen et al., 2011). After the co-incubation, parasites and HT29 cells were immunostained with antibody against the trophozoite-surface antigen TSA417 (red, specific for *Giardia*) and DAPI (blue). TSA417 is a member of VSP family and therefore its expression may not be uniform in by all trophozoites. However, in the current study the parasites were allowed to attach on HT29 cells for short period of time (30 min) and the immunostaining with anti-TSA417 was carried out after the cells were fixed in paraformaldehyde. Results shown in **Figure 3A** indicate that the trophozoites (red) attached to the HT29 cells (blue). Analysis also reveals that nystatin (27 μM) and oseltamivir (20 μM) inhibit this attachment significantly. For quantitative purposes, trophozoites (red) attached to HT29 cells were counted. Approximately, 50-100 co-cultured cells were examined from 5-10 different fields to identify attached cells. **Figure 3B** demonstrates that the trophozoite attachments are inhibited by nystatin, oseltamivir, and MBCD. However, oseltamivir is more potent than the other two LR inhibitors. Myriocin, an inhibitor of serine-palmitoyltransferase (SPT), has no effects on the attachment.

### (ii) Isolation and characterization of EVs: Nystatin and oseltamivir reduce the size of exosomes-like particles

**Figure 4:**
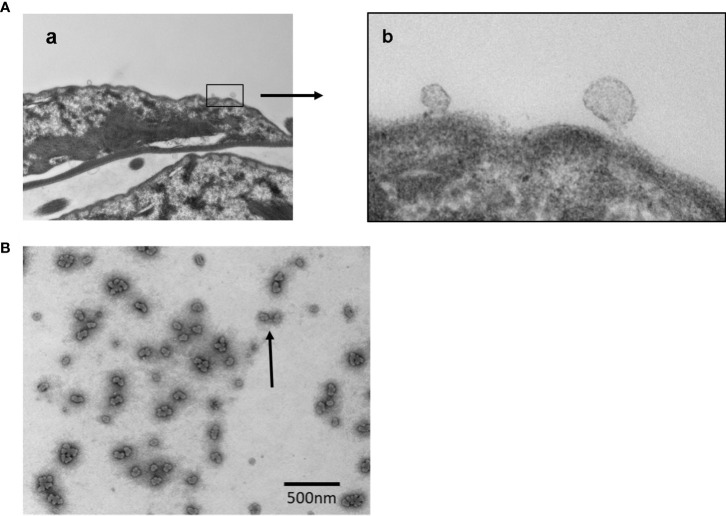
Transmission Electron Microscopy (TEM) analysis of vesicle formation and the images of released vesicles by *Giardia* trophozoites. Panel **(A)** Images showing **(a)** the budding of vesicles in lower magnification; **(b)** vesicles generation in higher magnification. Panel **(B)** Vesicles (LVs and SVs) released by trophozoites were isolated as mentioned in Materials and Methods and subjected to negative staining before the analysis by TEM. Bar: 500 nm.

EVs are released by *Giardia* and other parasites for inter-cell communications and host interactions (de Souza and Barrias, 2020). TEM analysis of EVs released by trophozoites. **Figures 4A** **, B** demonstrate the blebbing of vesicles from the plasma membranes. 

**Figure 5:**
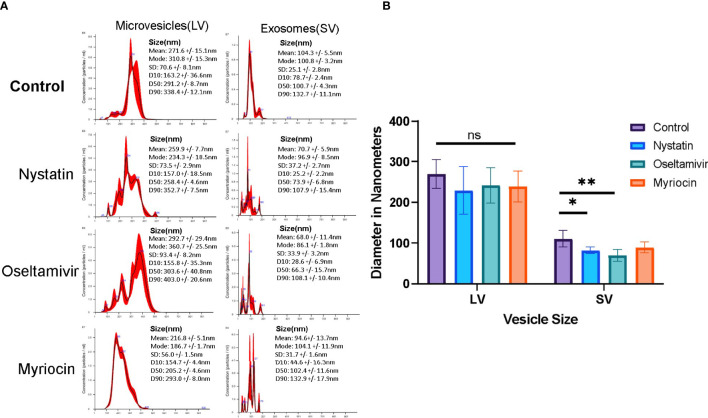
Characterization of vesicle population released by *Giardia*. **(A)** Large vesicular particles (LVs) and small vesicular particles (SVs) were isolated (Gavinho et al., 2020) and identified using a NanoSight. Results show a comparison of LVs and SVs from control, nystatin (27 μM), oseltamivir- (20 μM) and myriocin (20 μM) treated *Giardia* trophozoites. **(B)** NanoSight statistical comparisons of the sizes between LVs and SVs from data acquired from nanoparticle tracking analysis (NTA). The data is representative of at least three independent experiments and represented as ± SD. *P < 0.05; **p<0.001; ns, non-significant.

For additional characterization, EVs were isolated following the method of Gavinho et al. (2020), with some modifications and analyzed by NTA. Nystatin (27 μM) and oseltamivir (20 μM) were used to determine whether they would influence the release of vesicles. Myriocin (20 μM) was included as a negative control (De Chatterjee et al., 2015). EV secretion by control and drug-treated trophozoites was carried out for 3 h at 37°C, in nutrient-supplemented PBS, as described in Materials and Methods section, to collect the EVs for NTA. Cell viability (control and treated) was tested during the incubation period by flow-cytometry analysis after staining with propidium iodide (**Figure S1**). The NTA data indicate that *Giardia* produced two types of EV particles: LVs (mean size ~270 nm, microvesicle-like particles) and SVs (mean size ~100 nm, exosome-like particles) (**Figure 5**). Results also show that the size and pattern of SV particles are drastically altered by nystatin and oseltamivir (**Figure 5A**). Statistical analysis demonstrates that oseltamivir-treated cells show a significant decrease in size (p<0.01), followed by nystatin-treated *Giardia* (p<0.05) (**Figure 5B**). The effect of myriocin is not significant. Interestingly, these compounds did not show substantial effects on LVs.

### Proteomic analyses of lipid rafts, large vesicles, and small vesicles

To further understand the interplay between membrane LRs and EVs in *Giardia*, we conducted a proteomic analysis. gLRs were isolated by density gradient centrifugation, as described by De Chatterjee et al. (2015). Trophozoites were treated with nystatin (27 μM) and oseltamivir (20 μM) and labeled with CTXB-horseradish peroxidase (HRP), lysed in 1% Triton X-100 buffer, and subjected to OptiPrep gradient centrifugation. Fractions were collected from the top of the gradient and the GM1-enriched fractions were identified as previously described (De Chatterjee et al., 2015). Fractions 3–5 (from the top) were considered as LR fractions and subjected to proteomic analysis along with the non-LR fraction (Fraction 7) for comparison. LVs and SVs were isolated as described in the Materials and Methods section (Gavinho et al., 2020), before conducting a proteomic analysis, essentially following previously described methodologies (Szempruch et al., 2016; Ribeiro et al., 2018; Cortes-Serra et al., 2020; Cronemberger-Andrade et al., 2020).

**Figure 6:**
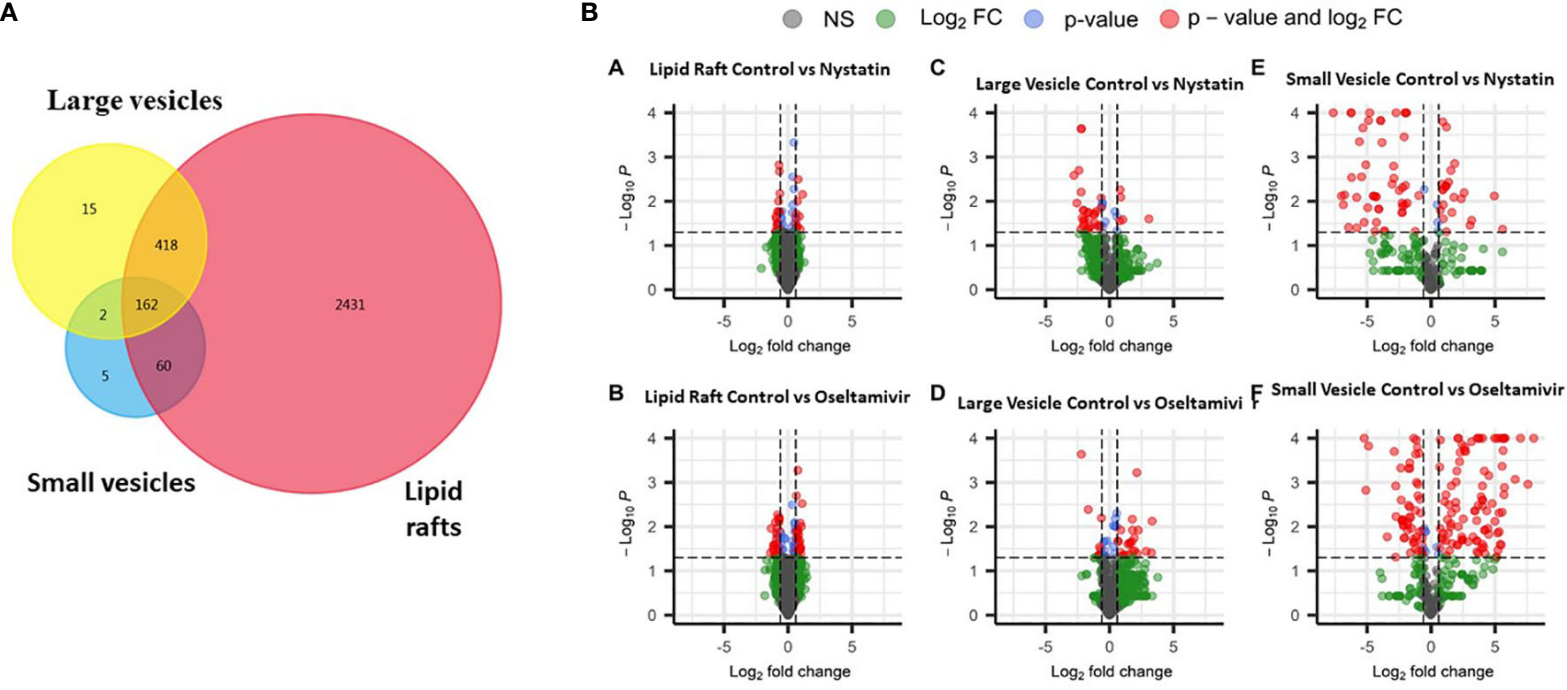
Proteomic comparison of rafts, LVs and SVs. **(A)** Venn diagram showing the distribution of proteins in three different components. **(B)** Volcano plots showing differentially expressed proteins between control and treatments with nystatin (27 μM) and oseltamivir (20 μM). Comparisons are shown in graphs a’ (raft control vs nystatin); b’ (raft control vs oseltamivir); c’ (LVs control vs nystatin), d’ (LVs control vs oseltamivir); e’ (SVs control vs nystatin) and f’ (SVs control vs oseltamivir). Volcano plots show –log10 P-values from the normalized proteomics data exported from Scaffold LFQ vs. log2 foldchange (FC) across each contrast **(A–F)**. Thresholds set were p-value ≤.05 and Log2FC ≤ -1.5 or ≥ 1.5.

**Figure 7:**
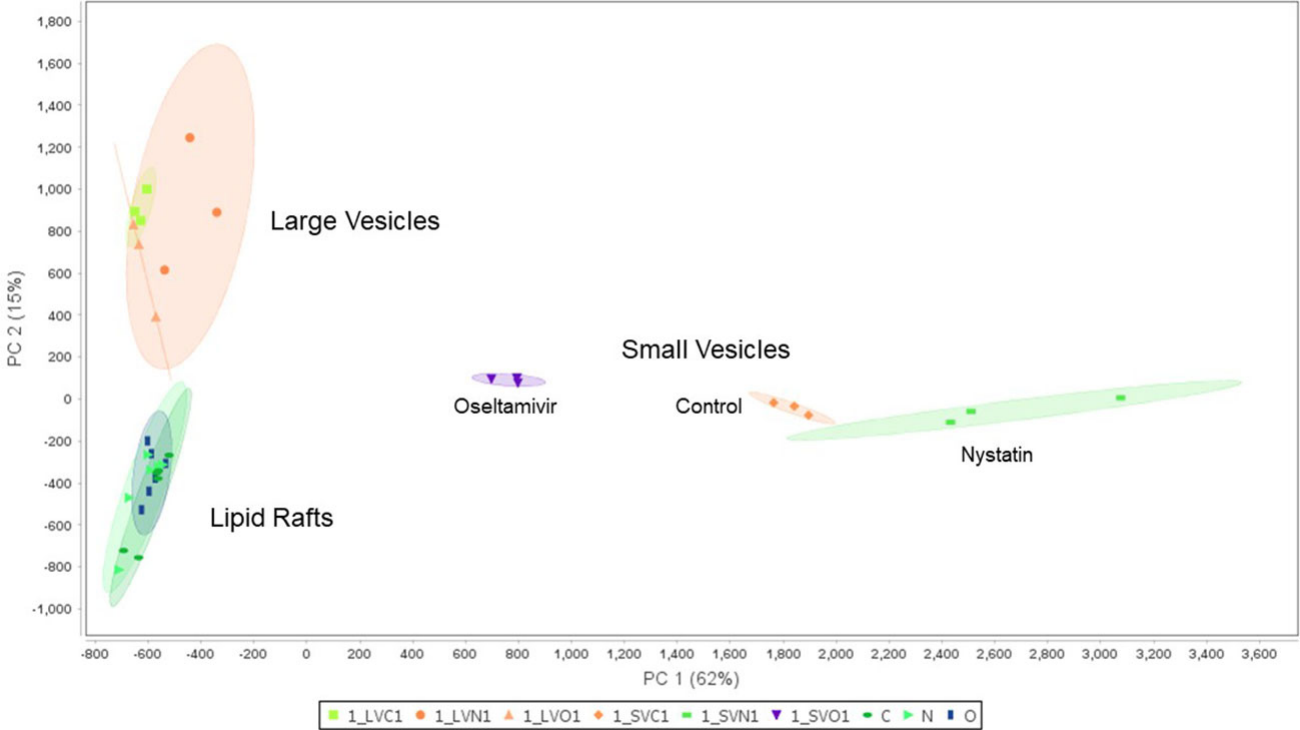
Principal component analysis (PCA) of proteomic data between the lipid rafts fractions in the control, nystatin and oseltamivir, the large vesicle fractions in the control, nystatin and oseltamivir, and the small vesicle fraction in the control, nystatin and oseltamivir.

**Figure 6** compares the proteomic profiles of LRs, LVs, and SVs. The Venn diagram shows that ~640 proteins are shared among the LRs, LVs, and SVs (**Figure 6A**). While only 418 proteins are shared between LRs and LVs, 60 proteins are common between LRs and SVs, and 162 proteins are shared by all three. Nystatin- and oseltamivir-induced changes in proteomic profiles are shown by the volcano plots (**Figure 6B**), and the original data are presented in **Tables S1**, **S2**. The analyses show the changes of protein expression in LRs, LVs, and SVs post-treatment. The drug treatments cause the up- and down-regulation of many proteins in all three components. However, most changes in the proteome profile (in comparison with untreated control) are observed in SVs (**Figure 6Be’, f’**). Moreover, oseltamivir treatment is more effective than nystatin in altering the proteome profile. This is in good agreement with our NTA results (**Figure 5**). Similar results with oseltamivir and LRs are also reflected in the principal component analysis (PCA) shown in **Figure 7**. The PCA analysis reveals that SVs are separated from LRs and LVs, and their protein profiles are significantly affected by nystatin and oseltamivir. On the other hand, although LVs are separated from LRs, their protein expression profile is affected slightly by either of these two drugs.

**Figure 8:**
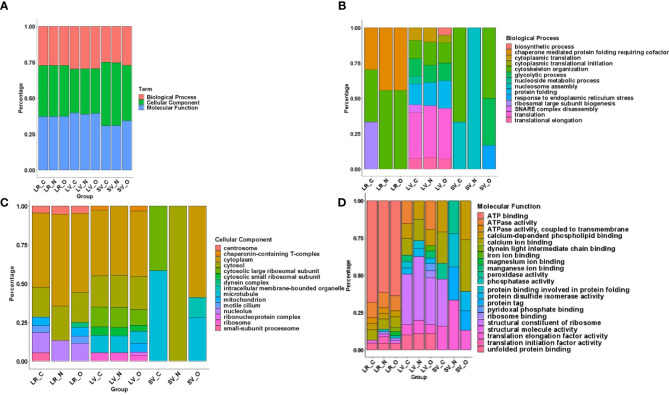
Protein Enrichment using DAVID was executed on RAW data across all groups including Lipid Raft: Control (LR_C), Nystatin (LR_N), Oseltamivir (LR_O), Large Vesicle: Control (LV_C), Nystatin (LV_N), Oseltamivir (LV_O) and Small Vesicle: Control (SV_C), Nystatin (SV_N), Oseltamivir (SV_O) as seen in panel **(A)** This was to determine their **(B)** Biological Process **(C)** Cellular Component and **(D)** Molecular Functions. Charts are normalized to a 100% for a more indicative representation of changes in process, component or function across fractions and treatment.

**Figure 9:**
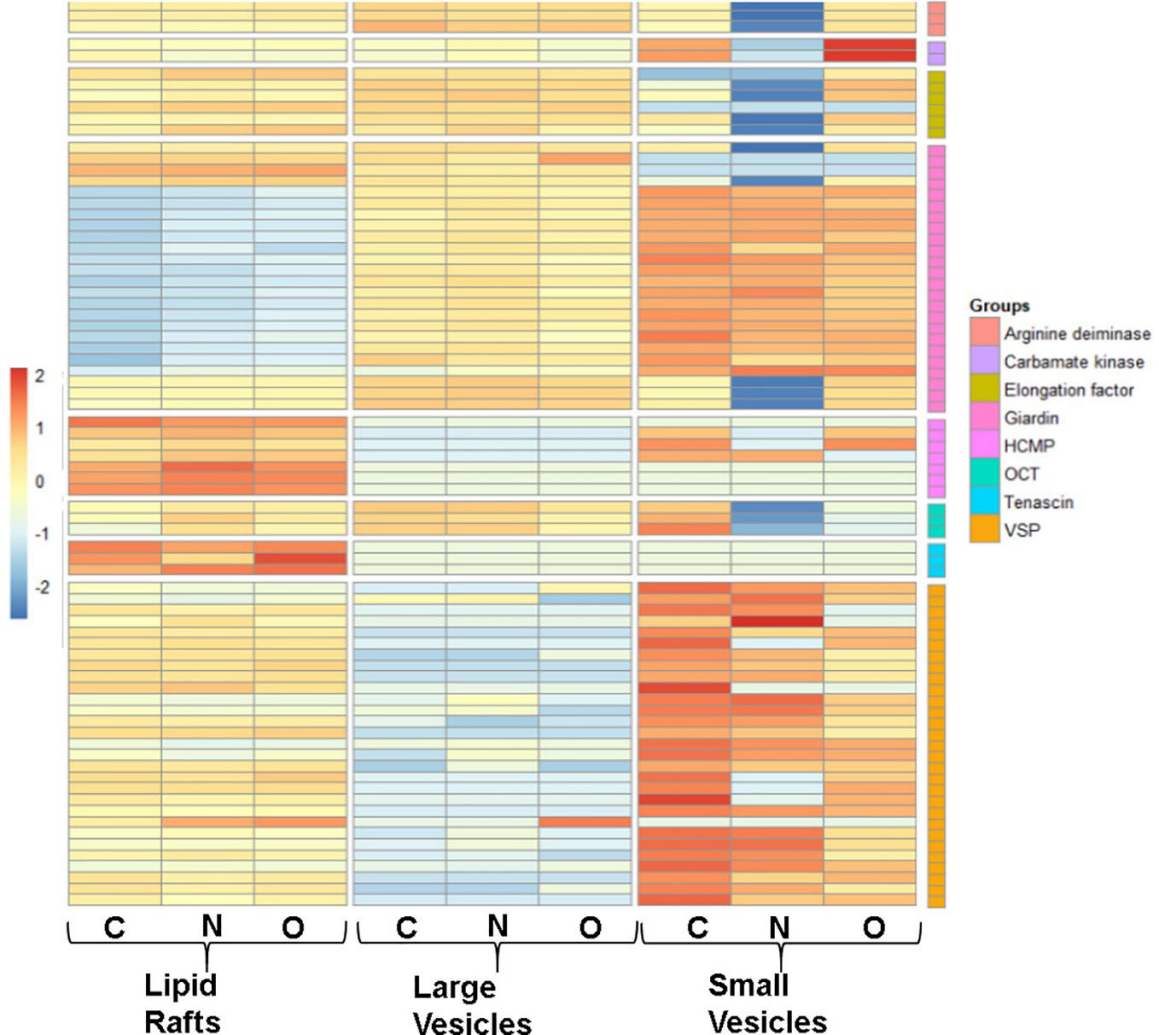
Differentially expressed heatmap patterns of potential giardial virulence factors such as arginine deiminase (AD), carbamate kinase (CK), elongation factor (EF), giardin, high cysteine membrane protein (HCMP), ornithine carbamoyltransferase (OCT), tenascin, and variant surface protein (VSP). Each heatmap contains proteins between control **(C)** and treatments with nystatin **(N)** (27 μM) and oseltamivir **(O)** (20 μM) within isolated lipid rafts, large vesicles and small vesicles. Protein samples included have a p-value ≤.05 between normalized data in scaffold and have been transformed to a Z-score for visual simplification.

**Figure 10:**
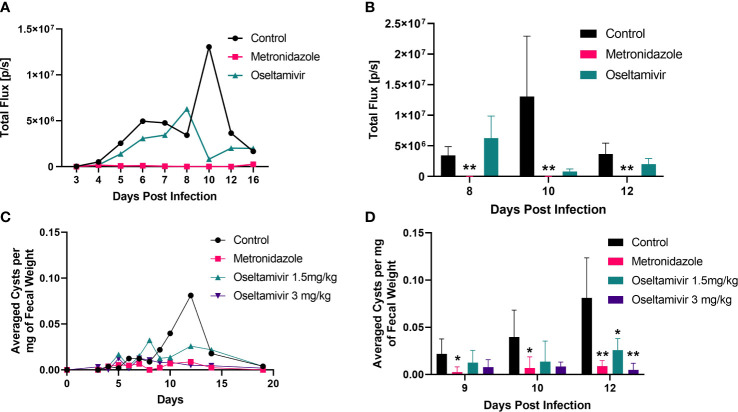
Inhibition of *Giardia* infection by oseltamivir in laboratory mice. Three to four-week-old mice (C57BL/6) were infected with luciferase-expressing trophozoites as detailed in Materials and Methods and tracked for colonization in the intestine followed by counting cysts in feces. **(A)** The treatment was initiated with oseltamivir (1.5 mg/kg) after 48 h of infection. Metronidazole (3.0 mg/kg) was used as a positive control. Bioluminescent signals from mice intestine (5 per group) were imaged through IVIS Lumina III *In Vivo* Imaging System (Perkin Elmer) to track the colonization (expressed total flux) are shown. **(B)** Statistical analysis of the bioluminescent experiments shown in Panel **(A, C)** Cyst counts in feces belonging to four different group of mice: control, metronidazole 3mg/kg, and oseltamivir at 1.5 mg/kg and 3mg/kg. Fecal counts were normalized by dividing the total amount of cysts identified to the total fecal weight collected. **(D)** Statistical significance of the reduction in fecal excretion of *Giardia* cysts by nystatin and oseltamivir. A total of five mice were used between each group. Nonparametric T-test was conducted on peak days of infection. *p < 0.05, **p < 0.01.

To identify proteins that are partitioned in gLRs, LVs, and SVs, we ran an enrichment analysis to classify them pre-and post-treatment. The stacked bar in **Figure 8** shows (A) percentage of enrichment; (B) biological process; (C) cellular component, and (D) molecular function. **Table S3** shows the enrichment statistics and identifies the proteins belonging to each enrichment class. For example, in the category of proteins that participate in biological processes, the LR inhibitors nystatin and oseltamivir cause drastic changes in ribosomal large-subunit biogenesis (**Figure 8B**). Additionally, enrichment analysis was carried out on the protein overlaps between LR, LV, and SV groups (**Table S4**). In LVs, the proteins involved in SNARE-complex disassembly are lost after treatment with the drugs. In SVs, while the proteins involved in cytoskeletal organizations are depleted by nystatin, the levels of ER stress-respond proteins are increased by oseltamivir (**Figure 8B**). In the cellular-component analysis (**Figure 8C**), we find no ribosomal proteins present in SVs. Microtubule-related proteins are decreased by oseltamivir and disappear after treatment with nystatin. An increase in cytosolic and cytoplasmic proteins in SVs by nystatin and oseltamivir treatment is also visible (**Figure 8C**). Finally, in the molecular-function category in SVs (**Figure 8D**), the proteins involved in peroxidase activity are not visible in treated samples. Because of the vast number of proteomic changes that occur after drug treatments, we reduced the scope of the proteomic profile comparisons and focused on potential virulence factors that have been identified in the literature and are likely to participate in host-parasite interactions (de Souza and Barrias, 2020). **Table S5** is the list of virulence factors identified in LRs, LVs, and SVs by the proteomic analysis. **Table S6** documents similarities and differences between the virulence-protein groups. To further illustrate this, we constructed a heatmap with virulence proteins that have been significantly up- or down-regulated when compared with their respective controls **(** **Figure 9**, **Table S7** **).** The PSM (peptide-spectrum match) values were normalized into Z-scores to better visualize changes within the data. The heatmap is divided into LRs, LVs, and SVs with and without the drug treatments, and is clustered into eight virulence factor groups. Results show that SVs express higher levels of giardins, CKs, and VSPs compared to LRs and LVs. The analysis also reveals that the LRs express higher levels of HCMPs and tenascins. On the other hand, two isotypes of delta giardins (accession numbers: A8BEZ6 and E1F6V5) are expressed ~3-4 fold higher in LVs compared to LRs and SVs. Interestingly, although nystatin and oseltamivir alter the expression of many of these proteins in LRs, LVs, and SVs, the effects of these two drugs are more robust on SV proteins. Both nystatin and oseltamivir reduced the expressions of most of the potential virulence factors, and oseltamivir is found to be more effective than nystatin (**Figure 10**).

### Oseltamivir reduces *Giardia* infection in mice

Because oseltamivir is more potent than nystatin in disassembling gLRs, blocking the attachment of trophozoites, and reducing the size of the exosome-like SVs, we decided to test this compound on mice infected with *Giardia*. Briefly, 3–4-week-old mice (C57BL/6) were infected with 1 x 10^7^ luciferase expressing (PNT5-Luc+) trophozoites (Pan et al., 2009) as described in Materials and Methods. Barash et al. (2017) also used luciferase-expressing *Giardia* (WB isolate) to locate high density foci of parasite in the small intestine of mice and gerbils. In the current investigation, the treatment with oseltamivir was initiated at day 2 post infection with gavage and continued until day 13. Metronidazole was used as a positive control. To assess the colonization of *Giardia* trophozoites in the small intestine, luciferin substrate (Gold Bio) was injected intraperitoneally, and the sedated mice were imaged through the IVIS. **Figure 10A** shows that the bioluminescent signals captured by IVIS as early as day 5 of post-infection, which increased steadily until day 10 before it declined by day 12. (Panel A). Other laboratories have reported that *Giardia* (both WB and GS) infection become significantly higher by day 4 or 5 of post-infection and peeks around day 7 to 10 (Solaymani-Mohammadi and Singer, 2011; Bartelt et al., 2013; Barash et al., 2017). The differences of infection profile in published work and in the current study could be due to the use of high doses of antibiotics (Crouch et al., 1990) and the changing of microbiome status in mice (Maarten's et al., 2021). There could be other host factors that might influence the colonization timing of trophozoites in the small intestine of mice used in this study. Nonetheless, the use antibiotics was necessary to establish infection by WB strain (Singer and Nash, 2000).

The focus of our experiment was to see if oseltamivir could reduce the colonization of parasite in mice. A cohort of five mice was imaged and the average of intensity (in total flux) was plotted against the days of post-infection. However, there were substantial variations in bioluminescent signals of individual mice (control and 1.5 mg/kg oseltamivir-treatment). The variation in bioluminescent signaling was also observed by Barash et al. (2017). **Figure 10A** shows that oseltamivir at 1.5 mg/kg reduced the bioluminescent signals emanating out of the small intestine effectively but was not statistically significant. However, this bioluminescent data gave us the guidelines to carry out cyst experiments described below.

For measuring the cysts released by infected animals, fecal materials from untreated and treated mice were collected and cysts were isolated by sucrose-floatation technique (described in Materials and Methods) and counted. Oseltamivir administered in a dose of 3 mg/kg shows a significant decrease of cysts in stool samples of mice (**Figures 10B** **, C**). The efficacy of oseltamivir is comparable to that of metronidazole, which was also administered at 3 mg/kg. Oseltamivir at 1.5 mg/kg fared significantly better than the control, showing a sharp decrease during peak infection at day 12. We found the variations of cysts counts of individual mice were minimum compared the bioluminescent study (**Figure 10A**) described above. The statistical analysis demonstrated that significant infection reduction (assessed by counting the cysts released by animals) was observed on day12 of the infection (**Figure 10D**). After day 12, the parasite load diminishes because most *Giardia* infections are self-limiting.

## Discussion

*Giardia, Entamoeba*, and *Cryptosporidium* are prominent members of a group known as “intestinal parasites”. However, they follow distinct strategies to produce infections in humans. For example, cysteine proteases and Gal/GalNAc-lectins produced by *Entamoeba* allows this parasite to disrupt the mucosal layer and invade the colonic epithelial cells. In *Cryptosporidium*, proteins such as galactose/*N*-galactosamine-specific lectin and the release of *T. gondii*-SAG1 homologue (Martínez-Ocaña et al., 2020) facilitate the attachment to host cells, followed by invasion (Wanyiri and Ward, 2006). On the other hand, *Giardia* is a strictly a noninvasive parasite that depends upon successful attachment to intestinal cells to establish the infection and induce cyst formation. Although it has been thought that the adhesive disc and flagellar motility in *Giardia* are essential for attachment (Holberton, 1974; Chavez and Martinez-Palomo, 1995; House et al., 2011), the participation of surface membrane-associated phosphomannosyl-binding lectins have also been proposed (Ward et al., 1990). MBCD, a common lipid raft inhibitor, has been shown to inhibit the attachment of *Giardia* on cultured Caco-2 cells, indicating that LR domains might support the adherence of this parasite on host cells (Humen et al., 2011). Our results demonstrate that the disassembly of gLRs with nystatin and oseltamivir inhibit the attachment of trophozoites on HT-29 cells (**Figure 3**) by damaging the plasma membrane (**Figure 3**). However, it is not clear at this point whether these compounds only target membrane rafts or have other targets.

We have previously demonstrated that the disruption of gLRs blocks encystation and cyst formation in culture (De Chatterjee et al., 2015). Thus, it is likely that cholesterol, sphingolipids, and protein-enriched raft microdomains function as “molecular switches” that relay extracellular signals to trophozoites for various cellular functions including the attachment, morphologic transformation to cysts and regulation of the host-parasite interactions. To further validate this hypothesis, we isolated and conducted a proteomic analysis of gLRs, which revealed that a large pool of proteins is partitioned into the raft domains. The pool includes proteins of biological processes, cellular components, and molecular functions (**Figure 8**). In addition, proteins, known to be virulence factors of *Giardia* (Ma’ayeh et al., 2017; Coelho and Singer, 2018; Ortega-Pierres and Argüello-García, 2019) are also present in gLRs (**Figure 9**), which raises the possibility that raft domains could be linked to giardial pathogenesis.

According to the current infection model, putative virulence factors are secreted by trophozoites during the host-*Giardia* interactions and interact with host cells (Ma’ayeh et al., 2017). These factors are present in “secretome” and include various iosotypes of ADIs, CKs, giardins, VSPs, EF-1s, OCTs, HCMPs, tenascins, and proteases. The immunodominant and glycosylated proteins are also secreted in the co-culture medium (Dubourg et al., 2018; Ortega-Pierres and Argüello-García, 2019). A transcriptomic analysis of Caco-2 cells infected with GS isolate revealed that *Giardia* induced the activation of the inflammasome, production of cytokines as well as activation of other signaling molecules and transcription factors. *Giardia* infection also causes changes in the metabolic status of intestinal epithelial cells (IECs) and altered the structure of tight junction and microvilli (Ma’ayeh et al., 2018).

Eukaryotic cells secrete EVs containing lipids, proteins, and nucleic acids. Although EVs are important for maintaining cell-to-cell communication, in a disease state, EVs could also function as pathogenic determinants. For instance, EVs released by malignant brain cells have been shown to promote the progression of brain metastases (Sirkisoon et al., 2022). Likewise, the parasitic cells use EVs to transfer cargoes in modulating the host immune system (Sabatke et al., 2021). It has been reported that giardial multicellular vesicles (MVs) are absorbed by dendritic cells, resulting in increased allostimulation (Evans-Osses et al., 2017). In other reports, EVs released by *Giardia* have been shown to affect the TLR2 and NLRP3 inflammasome pathways and proinflammatory immune responses in mouse macrophages *via* the MAPK, AKT, and NF-κB pathways (Zhao et al., 2021a; Zhao et al., 2021b).

In this article, we have shown that *Giardia* spontaneously releases two types of vesicles: large and small. More importantly, the LR inhibitors (i.e., nystatin and oseltamivir) reduced the size of small vesicles significantly. However, there was no significant change in the size of large vesicles (microvesicles-like particles) (**Figures 5A** **, B**). An earlier report demonstrated that MBCD, a common raft inhibitor, blocked the production of giardial microvesicles through a possible connection between the raft assembly and vesicle biogenesis (Evans-Osses et al., 2017). Regarding the origin of exosomes, studies with mesenchymal cells have revealed that exosomes are derived from endocytosed LRs (Tan et al., 2013), and lipids as well as proteins in LRs play significant roles in EV and exosome biogenesis (Skryabin et al., 2020).

Our proteomic analysis indicates that a substantial number of proteins are shared among LRs, LVs, and SVs. Interestingly, nystatin and oseltamivir show maximum effects on protein profiles in SVs, and oseltamivir is more effective than nystatin, possibly because both drugs have different targets. While oseltamivir targets GM1 synthesis/transport, nystatin binds with cholesterol (De Chatterjee et al., 2015). Since *Giardia* lacks the machinery to synthesize GM1 or other sphingolipids *de novo*, most of these lipids are imported from outside *via* clathrin-dependent- and independent-endocytic pathways and through the LRs (Hernandez et al., 2008). As exosomes are derived from LRs (Tan et al., 2013) and are likely to be enriched in GM1, it is possible that they are more sensitive to oseltamivir. Further analysis (**Figure 7**) revealed that proteins involved in various cellular, biological, and molecular functions are differentially affected by nystatin and oseltamivir in LRs, LVs, and SVs, suggesting the targets of these compounds may vary from rafts to vesicles. Interestingly, giardial virulence factors that are present in LRs, LVs, and SVs (**Figure 8**), are affected by these two drugs but again the effects are much more noticeable in SVs, further supporting the notion that exosomes in *Giardia*, like mammalian cells, originate from endocytosis at the LRs of the plasma membrane as proposed by other investigators (Tan et al., 2013; Skryabin et al., 2020). In general, while microvesicles are produced by membrane fusion, exosomes are generated from the invagination of late-endosomal membranes. This process results in intraluminal vesicles that mature into multivesicular bodies (MVBs), which can then bind to the membrane and release its cargo of exosomes (Minciacchi et al., 2015). The disruption of ceramide or cholesterol synthesis can alter the exosome composition by decreasing common exosome markers like flotillins and decreasing EGFR (epidermal growth factor receptor) in both the MVBs and exosomes (Gauthier-Rouvière et al., 2020). This process required Rab31, which is recruited in cholesterol and ceramide-rich membranes while interacting with flotillins and inducing EGFR packing (Gauthier-Rouvière et al., 2020; Kenific et al., 2021). In *Giardia*, however, the mechanism of microvesicle blebbing and exosome generation requires further experimentation since secretory pathways, such as the endosomal sorting complex required for transport (ESCRT), are not fully elucidated, although ESCRT associated proteins such as Vps4a, Rab 11 etc. are present in this organism (Moyano et al., 2019). Recently, using the high-end electron microscopy techniques, Midlej et al. (2019) has demonstrated that the peripheral vacuoles (PVs) in trophozoites exhibit morphological characterizations of MVBs (mean diameter ~50 nm) consisting of intralumenal vesicles (please see Benchimol and de Souza, 2022 for review). These reports support the idea that *Giardia* has the cellular machineries to generate both microvesicles and exosomes like higher eukaryotes.

In *Giardia*, virulence factors are known to modulate the immune system and cause damage to host cells (Coelho and Singer, 2018; Dubourg et al., 2018; Ortega-Pierres and Argüello-García, 2019; Zhao et al., 2021a; Zhao et al., 2021b). In our study, we found that the expressions of these virulence factors are affected after treatment with nystatin and oseltamivir. For example, ADI, CK, and OCT, which are engaged in arginine metabolism pathways, and suppress nitric oxide synthesis by the host intestinal cells (Stadelmann et al., 2012; Stadelmann et al., 2013) are altered by these two drugs (**Figure 9**). The significant changes of VSPs, HCMPs, giardins, and EF-1α expressions are also visible, indicating that that these two raft disruptors have broader effects on virulent protein secretion by *Giardia*. VSPs and HCMPs, which are abundant in *Giardia*, and present in the parasite’s membranes and vesicles are also affected. The expressions of VSP9B10A, a cysteine protease (Argüello-García and Ortega-Pierres, 2021) and EF-1α, which is a nuclear protein in *Giardia* (Skarin et al., 2011) are altered by nystatin and oseltamivir, especially in SVs (**Figure 9**).

We have previously reported that oseltamivir inhibits encystation and cyst production in culture by disassembling LRs (De Chatterjee et al., 2015). In the current study, we have shown that oseltamivir disrupts plasma membrane structures, inhibits attachment of the parasite, alters exosome sizes, and changes the overall proteomic composition of gLRs, LVs, and SVs (**Figures 3** **–** **9**). These results encouraged us to examine if oseltamivir could be used as an anti-giardial agent. As previously mentioned, oseltamivir is a known anti-H1N1 flu drug, and its potential use against *Giardia* must strengthen the “drug repurposing” effort (Pushpakom et al., 2019). Furthermore, metronidazole, a commonly prescribed drug to treat giardiasis, is a genotoxic agent and a potential carcinogen, and its frequent use gives rise to drug-resistant giardiasis (Riches et al., 2020). Thus, we used a mouse model to evaluate the effects of oseltamivir in *Giardia* infection. Mice were infected with luciferase-expressing *Giardia* trophozoites, and infection was tracked for 13 days with the help of IVIS **(** **Figure 10A** **).** The number of cysts in the mouse fecal samples were collected and counted as described in the Methods Section. For the treatment, two doses of oseltamivir were used —i.e., 1.5 and 3.0 mg/kg body weight of mice. At 1.5 mg/kg, a significant drop of infection was observed at day 13, when the infection was maximum (**Figure 10B**). Oseltamivir at 3.0 mg/kg—comparable to metronidazole (3 mg/kg), which was used as a positive control—reduced the cyst production completely **(** **Figure 10** **C** **).** This reduction allows for the possibility that oseltamivir, which targets raft assembly and vesicles synthesis, could be used as potential anti-giardial agent in the future.

**Figure 11:**
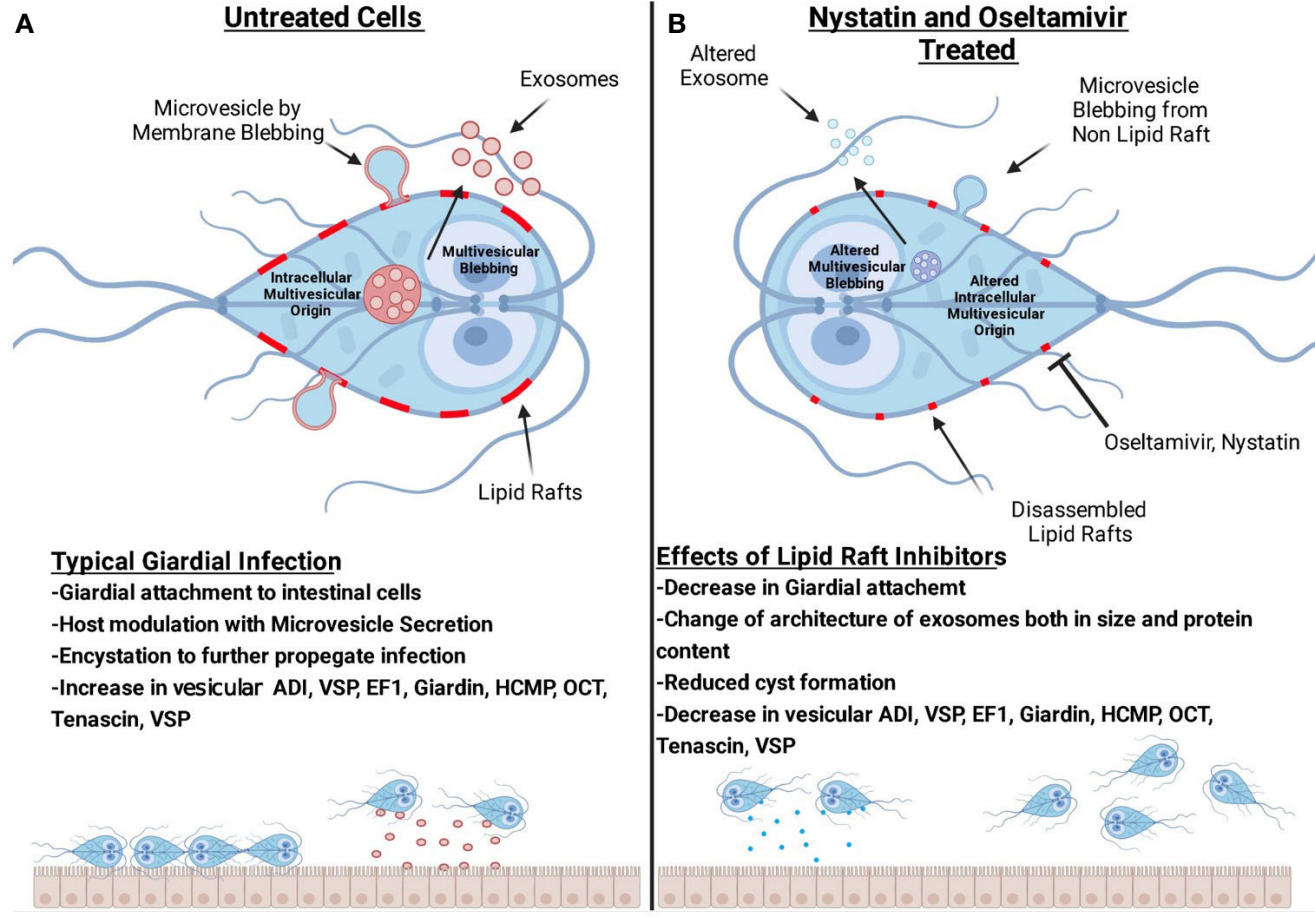
Hypothetical models representing the interplay among lipid rafts, microvesicles-like large vesicles, exosomes-like small vesicles and intestinal cells. Panel **(A)** shows the blebbing of large vesicles from raft- and non-raft membranes of trophozoites. The release of small vesicles is also shown. Our results suggest that lipid rafts support the attachment of trophozoites on host cells and produce infection. Vesicles are secreted by this parasite to modulate host response and the breakdown of intestinal cellular integrity. Panel **(B)** shows that both oseltamivir or nystatin disrupts rafts and affects large as well as small vesicles. The architecture, size, and composition of vesicles, specifically the small vesicles are severely affected by these compounds. Lipid raft disruptors reduce the overall number of virulence factors present in vesicles. Oseltamivir and nystatin disrupt raft domains and decrease the ability of *Giardia* to attach to intestinal cells, encyst and producing infection in mice. (The image was created with BioRender.com).

**Figure 11** is a comprehensive model demonstrating that rafts participate in the attachment of trophozoites to cultured intestinal cells. *Giardia* releases two types of vesicles: large microvesicles and small exosomes. Our proteomic analysis reveals that raft and vesicles contain proteins and enzymes, including the proteins known as giardial virulence factors. These proteins such as ADI, VSP, EF-1, giardin, HCMP, OCT, CK, and tenascins (**Figure 11A**) are shared among LRs, LVs, and SVs. Nystatin and oseltamivir block the attachment of trophozoites by disrupting LRs and reducing the size of SVs (**Figure 11B**). Although protein profiles in rafts, LVs, and SVs are altered by nystatin and oseltamivir, SVs are affected more compared to LVs. Finally, oseltamivir was found to be effective in reducing the parasite load in mice.

Our future goal is to synthesize and evaluate oseltamivir analogues on metronidazole-resistant *Giardia* both in culture and in a mice model. However, it is important to note that the effects of treatment with oseltamivir, and its active metabolites are indeed profound and complex. For example, administration of oseltamivir in mice challenged by a respiratory syncytial virus (RSV) that lacks a neuraminidase gene showed relief of symptoms and inhibition of viral clearance. These effects are correlated with decreased T cell surface GM1 that is regulated by the endogenous neuraminidase in response to viral infection. In addition, the administration of oseltamivir is associated with a significant reduction in pro-inflammatory cytokines such as interferon-gamma, interleukin-6, and tumor necrosis factor-alpha. Further, there has been speculation that reduction of antibody production and cytokine induction, as well as renal, metabolic, cardiac, and prolonged psychiatric disorders after oseltamivir use may be related to inhibition of the host’s endogenous neuraminidase. Yet, in the above case, clearance of the infectious agent was inhibited and these other off-target effects of oseltamivir and its active metabolite should be expected to inhibit clearance of an infection and not to aid in its clearance (Hama, 2016).

## Data availability statement

The datasets presented in this study can be found in online repositories. The names of the repository/repositories and accession number(s) can be found in the article/**Supplementary Material**.

## Ethics statement

The animal study was reviewed and approved by IACUC, University of Texas at El Paso.

## Author contributions

BG, SP, ICA, and SD designed the research. BG, ADC, BP, CE, and VE conducted the experiments. CV and AN were instrumental in performing d-STORM experiments. SRoyc and ADC acquired and analyzed confocal images. SRoy collaborated with BG in data acquisition and bioinformatic analysis. BG, SD, and SP analyzed the data and drafted the paper. ICA examined and commented on the data analysis and helped to complete the manuscript. All authors contributed to the article and approved the submitted version.

## Funding

This work was supported by a grant 1R21AI138061 from NIAID (NIH). The biochemical, molecular, and confocal microscopy experiments were conducted at the Biomolecule Analysis and Omics as well as Cellular Characterization and Biorepository Core Facilities at BBRC/UTEP supported by U54MD007592 (NIMHD/NIH). We also acknowledge support from the Building Scholar Grant (8RL5GM118969) to UTEP from NIGMS (NIH). TEM images were taken at the Molecular Microbiology Imaging Facility (Director: Dr. Wendy Beatty) of the Washington University, St. Louis, Mo. Drs. Brian Grajeda and Cameron Ellis were supported by BBRC/UTEP. Dr. Vanessa Enriquez was supported by a RISE grant from the NIGMS (5R25GM069621). dSTORM imaging facilities and analysis methods were developed under auspices of NIH/NIGMS 5P50GM085273-05. We acknowledge support for AN and CV from NIH/NIAID R01AI116894.

## Conflict of interest

The authors declare that the research was conducted in the absence of any commercial or financial relationships that could be construed as a potential conflict of interest.

## Publisher’s note

All claims expressed in this article are solely those of the authors and do not necessarily represent those of their affiliated organizations, or those of the publisher, the editors and the reviewers. Any product that may be evaluated in this article, or claim that may be made by its manufacturer, is not guaranteed or endorsed by the publisher.

## Supplementary material

Supplementary Figure 1 Testing the viability of *Giardia* trophozoites by flow cytometry. Live parasites (control and treated) were stained with the membrane-impermeant dye propidium iodide (5µg/ml) as described by Ruiz-Medina et al. (2019). Cells were collected in a flow cytometric tube, stained with PI, vortexed gently, and analyzed immediately with a flow cytometer (Cytomics FC500; Beckman Coulter, Miami, FL). A maximum of 10,000 events (cells) were collected for each sample, and data were analyzed using CXP software (Beckman Coulter, Miami, FL). Experiments were repeated three times and a representative analysis is shown here. Results show that more than 98% cells remain viable after the treatment with nystatin and oseltamivir. On the other hand, formaldehyde (positive control) killed more than 99% of cells during the same period of incubation.

Supplementary Table 1 Differentially expressed proteins processed through ScaffoldQ+ 4.8.2. Proteins with ap-value ≥.05 were considered significant. Students T-tests were run across all comparisons that are represented in their respective spreadsheets. Spreadsheets include the comparisons, which include proteins between control (C) and treatments with nystatin (N) (27 μM) and oseltamivir (O) (20μM) within purified lipid rafts (LRs), large vesicles (LVs) and small vesicles (SVs) as shown in . After a comparisons tab there are two concurrent spreadsheets which include an enrichment showing the GO annotation of up and down regulated proteins from the comparisons.

Supplementary Table 2 Proteomic output of Normalized Peptide Spectral Matches (PSM) from fractions of lipid rafts (LRs), large vesicles (LVs) and small vesicles (SVs) compared to their respective lipid raft disruptor treated group: oseltamivir (O) and nystatin (N). Student’s T-test was preformed between each group. Spreadsheets were used to construct volcano plots in R shown in **Figure 6**.

Supplementary Table 3 Database for annotation visualization integrated discovery (DAVID) enrichments of entire proteomic sets including the control and treated lipid rafts (LRs), large vesicles (LVs) and small vesicles (SVs). Each group is enriched in the biological process, cellular component and molecular function. Key terms in the tables include the GO Annotation enrichment (Term), proteins identified in the specific annotation (Count), the genes that were categorized into the GO annotation (Genes), and the p-value of the identification of the GO annotation (Benjamini). (**Figure 8**).

Supplementary Table 4 Significant proteins identified from the proteomic analysis of lipid rafts (LRs), large vesicles (LRs) and small vesicles (SVs). A student’s T-test was run across all comparisons that are represented in their respective tabs. The table includes the protein names, accession numbers and the averaged normalized weighted spectral counts as well as additional protein details. Concurrent spreadsheets show the protein overlaps between the groups. Overlaps comparisons additionally have enrichment analysis annotating how many proteins are found in each functional category.

Supplementary Table 5 Differentially expressed virulence proteins processed through ScaffoldQ+ 4.8.2.Proteins with a p-value≥0.05 were considered significant. A student’s T-test was run across all comparisons that are represented in their respective tabs. Spreadsheets include the respective comparisons which include proteins between control (C) and treatments with nystatin (N) (27μM) and oseltamivir (O) (20 μM) within isolated lipid rafts (LRs), large vesicles (LVs) and small vesicles (SVs).

Supplementary Table 6 All virulence proteins from proteomic analysis from lipid rafts, large vesicles and small vesicle were shown in one table. The table includes the protein names, accession numbers and the averaged normalized weighted spectral counts of the proteins. Concurrent spreadsheets show the distribution of these proteins between the three groups mentioned before as well as the gene name, organismal database and length.

Supplementary Table 7 The data output was used to generate the heatmap and contains averaged proteomic output of significant virulence proteins found between lipid rafts (LRs), large vesicles (LVs) and small vesicles (SVs) with treatment of oseltamivir and nystatin. Protein name, accession and normalized peptide spectral matches are mentioned (please see **Figure 9**).
