# Supplementary figures and images for: Giardial lipid rafts share virulence factors with secreted vesicles and participate in parasitic infection in mice
Source: Front Cell Infect Microbiol. 2022 Aug 23;12:974200. doi: 10.3389/fcimb.2022.974200 (PMC9445159; doi:10.3389/fcimb.2022.974200)

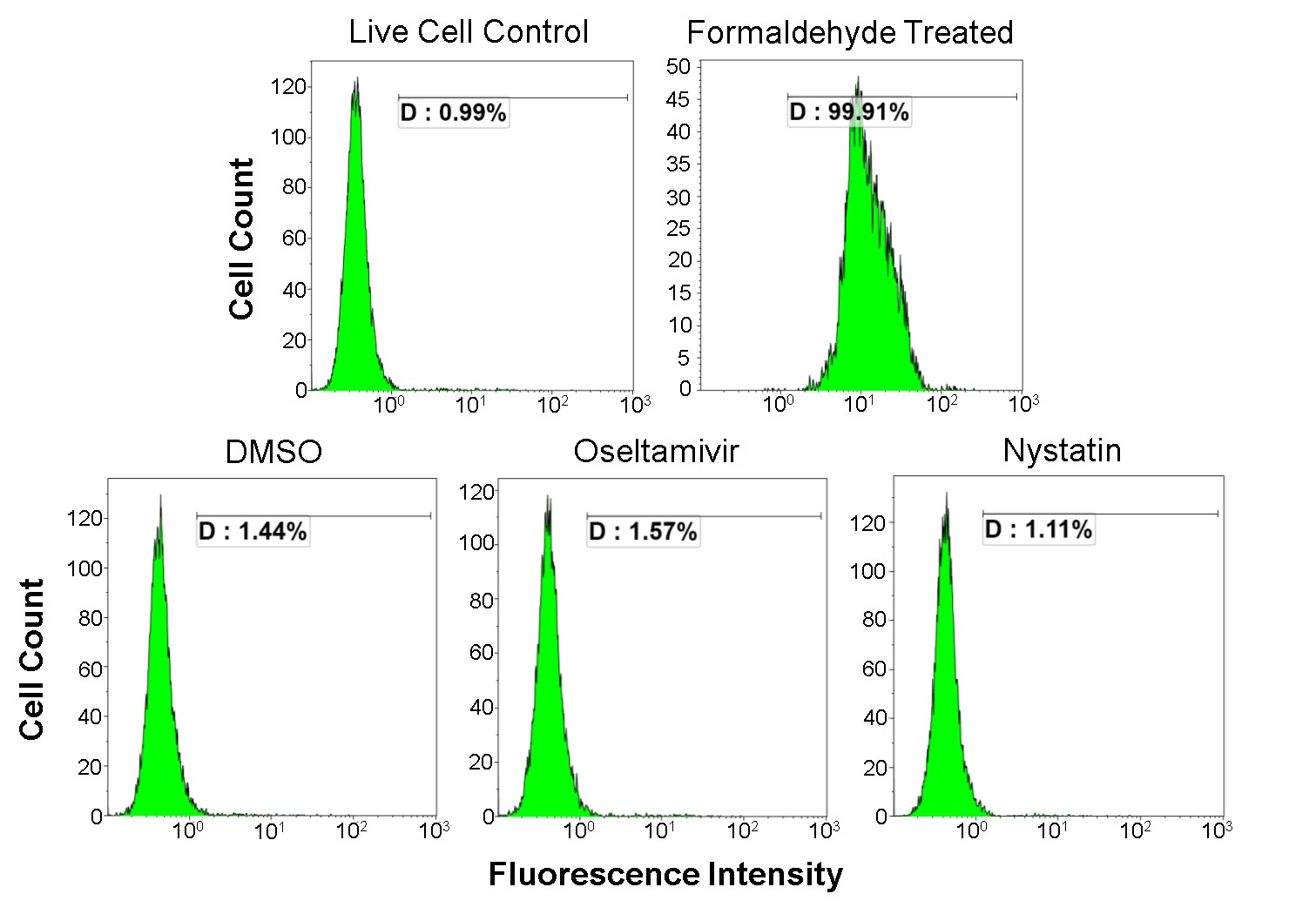

Supplement: Supplementary Figure 1 — Testing the viability of Giardia trophozoites by flow cytometry. Live parasites (control and treated) were stained with the membrane-impermeant dye propidium iodide (5µg/ml) as described by Ruiz-Medina et al. (2019). Cells were collected in a flow cytometric tube, stained with PI, vortexed gently, and analyzed immediately with a flow cytometer (Cytomics FC500; Beckman Coulter, Miami, FL). A maximum of 10,000 events (cells) were collected for each sample, and data were analyzed using CXP software (Beckman Coulter, Miami, FL). Experiments were repeated three times and a representative analysis is shown here. Results show that more than 98% cells remain viable after the treatment with nystatin and oseltamivir. On the other hand, formaldehyde (positive control) killed more than 99% of cells during the same period of incubation. [file Image_1.jpeg]
